# Social network size and personality traits independently and prospectively predict distress disorders and suicidal behavior in U.S. Army soldiers

**DOI:** 10.1017/S0033291722002082

**Published:** 2023-08

**Authors:** Charles T. Taylor, Laura Campbell-Sills, Ronald C. Kessler, Xiaoying Sun, Matthew K. Nock, Robert J. Ursano, Sonia Jain, Murray B. Stein

**Affiliations:** 1 Department of Psychiatry, University of California San Diego, La Jolla, CA, USA; 2 Department of Health Care Policy, Harvard Medical School, Boston, MA, USA; 3 Herbert Wertheim School of Public Health and Human Longevity Science, University of California San Diego, La Jolla, CA, USA; 4 Department of Psychology, Harvard University, Cambridge, MA, USA; 5 Center for the Study of Traumatic Stress, Department of Psychiatry, Uniformed Services University of the Health Sciences, Bethesda, MD, USA; 6 VA San Diego Healthcare System, San Diego, CA, USA

**Keywords:** Internalizing disorders, military, neuroticism, personality, social networks, suicide

## Abstract

**Background:**

Personality traits (e.g. neuroticism) and the social environment predict risk for internalizing disorders and suicidal behavior. Studying these characteristics together and prospectively within a population confronted with high stressor exposure (e.g. U.S. Army soldiers) has not been done, yet could uncover unique and interactive predictive effects that may inform prevention and early intervention efforts.

**Methods:**

Five broad personality traits and social network size were assessed via self-administered questionnaires among experienced soldiers preparing for deployment (*N* = 4645) and new soldiers reporting for basic training (*N* = 6216). Predictive models examined associations of baseline personality and social network variables with recent distress disorders or suicidal behaviors assessed 3- and 9-months post-deployment and approximately 5 years following enlistment.

**Results:**

Among the personality traits, elevated neuroticism was consistently associated with increased mental health risk following deployment. Small social networks were also associated with increased mental health risk following deployment, beyond the variance accounted for by personality. Limited support was found for social network size moderating the association between personality and mental health outcomes. Small social networks also predicted distress disorders and suicidal behavior 5 years following enlistment, whereas unique effects of personality traits on these more distal outcomes were rare.

**Conclusions:**

Heightened neuroticism and small social networks predict a greater risk for negative mental health sequelae, especially following deployment. Social ties may mitigate adverse impacts of personality traits on psychopathology in some contexts. Early identification and targeted intervention for these distinct, modifiable factors may decrease the risk of distress disorders and suicidal behavior.

## Introduction

Vulnerability to internalizing disorders (including anxiety, stress-related, and depressive disorders) and suicidal behavior is attributable to dispositional characteristics of the individual as well as features of the environment that influence exposure and reactivity to stressors – the central tenet of diathesis-stress models of psychopathology. Personality traits represent heritable, biologically based, and moderately stable (Smith et al., 2016; Wray, Birley, Sullivan, Visscher, & Martin, 2007) patterns of thought, behavior, and emotions that shape how individuals respond to their environment. Neuroticism is a personality trait characterized by the tendency to experience frequent and intense negative emotions in response to perceived stressors. It is one of the most extensively studied and validated dispositional factors that increases the risk for internalizing disorders and suicidal behavior (Barlow, Sauer-Zavala, Carl, Bullis, & Ellard, 2014; Kotov, Gamez, Schmidt, & Watson, 2010) and is increasingly positioned as a key treatment target (Sauer-Zavala et al., 2021).

Central to diathesis-stress models is the notion that aspects of the environment can potentiate or mitigate dispositional influences on psychopathology (e.g. Fox & Beevers, 2016). The social environment in particular has been shown to influence the risk for internalizing disorders (Saris, Aghajani, van der Werff, van der Wee, & Penninx, 2017) and suicidality (Holma et al., 2010), likely because social connections represent a fundamental human need that provide a sense of support and security (Baumeister & Leary, 1995). Perceptions of one's social environment may therefore modify the influence of dispositional factors such as neuroticism on the development of internalizing disorders and/or suicidal behavior. Personality and social factors, however, have largely been studied in separate lines of research conducted in community samples in which stressor exposure is unknown. It therefore remains poorly understood whether personality and the social environment operate independently, share substantial variance, or interact in conferring risk for subsequent internalizing disorders or suicidal behavior in a population with elevated stressor exposure. Our goal was to investigate these possibilities using data from the Army Study to Assess Risk and Resilience in Servicemembers (Army STARRS; Kessler et al., 2013a; Ursano et al., 2014).

The link between personality and psychopathology is often studied within the five-factor (Big Five) model of personality (McCrae & Costa, 2003). Neuroticism prospectively predicts major depression (Fanous, Neale, Aggen, & Kendler, 2007; Kendler, Gatz, Gardner, & Pedersen, 2006; Kendler, Kuhn, & Prescott, 2004), anxiety disorders (Zinbarg et al., 2016), and suicide (Fergusson, Woodward, & Horwood, 2000; Yen et al., 2009) in civilian samples. In military personnel, cross-sectional relationships were observed between higher neuroticism and posttraumatic stress and depressive symptoms measured post-deployment (Caska & Renshaw, 2013; James, Van Kampen, Miller, & Engdahl, 2013). Prospective studies are needed, however, because the neuroticism-psychopathology link attenuates considerably when accounting for baseline mental health symptoms (Jeronimus, Kotov, Riese, & Ormel, 2016).

Low conscientiousness and low extraversion are also associated with internalizing disorders (Kotov et al., 2010), whereas agreeableness and openness to experience are not (Kotov et al., 2010; Watson & Naragon-Gainey, 2014). Nevertheless, consideration of shared variance among personality dimensions is important because the Big Five traits are not completely orthogonal (Digman, 1997; Markon, Krueger, & Watson, 2005) and several traits may be regulated by common neurobiological systems (Wright, Creswell, Flory, Muldoon, & Manuck, 2019). Identifying unique prospective associations among the Big Five personality dimensions with internalizing disorders or suicidality could point to a specific trait or limited set of traits that offer the greatest explanatory power and may therefore be most profitable to target in universal screening and prevention efforts.

The social environment also influences vulnerability to psychopathology (Cacioppo & Cacioppo, 2018; Umberson & Montez, 2010), in part through regulating positive outcomes (e.g. engagement in pleasurable or meaningful activities) and negative outcomes (e.g. perceived availability of support during times of stress; see Berkman, Glass, Brissette, and Seeman, 2000). Prospective studies demonstrate that poor quality relationships (Teo, Choi, & Valenstein, 2013) and perceived social disability (Saris et al., 2017) increase the future risk of new onset and sustained episodes of anxiety and depression, as well as suicide attempts (Holma et al., 2010). Cross-sectional studies in military personnel demonstrate associations between perceived social network strength and a range of negative mental health sequelae post-deployment (James et al., 2013; Mitchell, Gallaway, Millikan, & Bell, 2012; Pietrzak et al., 2009), even when accounting for deployment stressors (Welsh, Olson, Perkins, Travis, & Ormsby, 2015). Prospective studies, however, are lacking.

Investigating the joint effects of personality and social network characteristics on internalizing disorder or suicidal behavior outcomes has not been done but could advance understanding of risk in several ways. First, if these risk factors share considerable variance, and one is found to largely explain the effect of the other, prevention efforts could focus on detecting and modifying the factor with greater explanatory power. Second, interaction models could reveal that the effects of one risk factor are potentiated or attenuated by the other factor. Because neuroticism is hypothesized to increase the risk for internalizing disorders and suicidality through inflated reactivity to stressors, having a strong social network may buffer the effects of stressor exposure through the provision of social support, encouraging cognitive reappraisal, engagement in positive affect-generating activities, and/or downregulation of stress-related biological systems (e.g. Berkman et al., 2000; Fredrickson, 2001, 2003; Hostinar, Sullivan, and Gunnar, 2014). Neuroticism may therefore only predict internalizing disorders or suicidal behaviors in the context of an impoverished social network. This type of knowledge would increase precision in identifying vulnerable individuals. Finally, prospective studies examining personality traits and social networks in military personnel are absent. Identifying modifiable risk factors in service members could offer actionable insights to inform prevention and early intervention efforts in the military, as well as other populations (e.g. first responders) who routinely encounter highly stressful situations.

The present study examined the main and interactive effects of personality traits and social network size on prospective mental health outcomes in two cohorts from Army STARRS (Kessler et al., 2013a; Ursano et al., 2014). The primary analysis was conducted using data from more than 4500 soldiers who completed surveys assessing internalizing disorders and suicidal behaviors shortly before deployment to Afghanistan and 3 and 9 months following their return from deployment. We focused on this cohort because all soldiers experienced known stressor exposure (deployment) and mental health outcomes were measured at discrete periods before and after that exposure. A supplementary analysis was conducted to examine whether effects observed in the primary analysis would extend over a longer period (5 years) and within a sample facing a variety of possible adjustment challenges, i.e., new recruits. The supplementary analysis was based on data from over 6000 soldiers who completed self-assessments of personality, social networks, and lifetime psychopathology prior to basic training and subsequently completed surveys evaluating internalizing disorders and suicidal behavior obtained on average 5 years later.

In defining the mental health outcomes of interest, we focused on the distress disorders subfactor of internalizing psychopathology [major depression, generalized anxiety disorder (GAD), and posttraumatic stress disorder (PTSD)] because these conditions are highly prevalent in service members (Kessler et al., 2014) and have been shown to share a common underlying structure (de Jonge et al., 2018). Suicidal behavior was also examined (as a separate outcome) because it is common among service members (Ursano et al., 2015) and is a high-priority area for the U.S. Army (Ursano et al., 2014). We hypothesized that among the personality variables, elevated neuroticism would show the most robust associations with distress disorders and suicidal behaviors. We also hypothesized that larger social networks would protect against distress disorders and suicidal behaviors. Given suggestions that only a few social connections may be enough to garner positive and mitigate negative health outcomes (Baumeister & Leary, 1995), or that too many social connections could be detrimental to mental health (e.g. through increasing demands on emotional and physical resources; Falci and McNeely, 2009), we also explored non-linear associations between social network size and mental health (see online Supplemental Materials). Finally, we hypothesized that personality (neuroticism in particular) and social network variables would interact such that the association between neuroticism and subsequent mental health outcomes would be attenuated in soldiers with the largest social networks.

## Methods

### Participants

Data were obtained from two Army STARRS cohorts. For detailed descriptions of the study design and procedures see online Supplemental Materials and (Heeringa et al., 2013; Kessler et al., 2013a; Ursano et al., 2014).

#### Pre/Post Deployment Study (PPDS)

Regular Army soldiers from three Brigade Combat Teams (BCTs) were recruited for a longitudinal panel survey before deploying to Afghanistan in 2012. Baseline evaluation occurred 1–2 months before deployment (T0). Follow-ups occurred within 1 month of return to the U.S. (T1), 3 months later (T2), and 9 months later (T3). The current models were tested within soldiers who completed surveys at all waves (*n* = 4645; 60.0% of the eligible sample of deployed soldiers). Response propensity and post-stratification weighting factors were developed and applied in PPDS analyses (Heeringa, West, & Berglund, 2010; see online Supplemental Materials). Sample demographic characteristics are presented in the online Supplemental Methods.

#### New Solider Study (NSS)

See online Supplemental Materials.

### Measures

#### Personality

The PPDS and NSS baseline surveys contained items adapted from previously validated self-report personality inventories (see Rosellini et al., 2017). We focus on the dimensions of neuroticism (7 items; PPDS T0 *α* = 0.82), agreeableness (3 items; PPDS T0 *α* = 0.54), openness to experience (4 items; PPDS T0 *α* = 0.37), extraversion (3 items; PPDS T0 *α* = 0.68), and low conscientiousness (2 items; PPDS T0 *α* = 0.54). See online Supplemental Materials for sample items, instructions, and scale descriptives.

#### Social networks

The social network scale comprised 4 items that assessed the size of different aspects of Soldiers' affiliative networks. Items were prefaced with, ‘How many people do you have in your personal life of the following sorts?’ and were rated on a 10-point scale referencing 0, 1, 2, 3, 4, 5, 6–10, 11–20, 21–30, or 31 or more people. Ratings were coded 0–9. Items referenced: ‘people you do things with, like watch TV together, go out for a drink or movie together, or play cards’; ‘people who you feel really close to’; ‘people who really care for you and would be there if you needed them’; and ‘family or friends who need you and rely on you for help when they need it.’ Exploratory factor analysis suggested a unidimensional structure (item-factor loadings: PPDS = 0.66–0.89). Items were summed to create a total social network score with higher scores reflecting larger social networks (range = 0–36; PPDS T0 *α* = 0.85).

#### Mental disorders and suicidal behaviors

Outcomes in the PPDS sample were composite indices reflecting presence *v.* absence of (1) any distress disorder [i.e. any diagnosis of PTSD, major depressive episode (MDE), or GAD] and (2) suicidal behavior (i.e. any suicide ideation, plan, or attempt) in the past 30 days. See online Supplemental Materials for a description of the survey assessment of mental disorders and suicidality.

#### Deployment stress

Soldiers in the PPDS cohort completed a Deployment Stress Scale (DSS; see Campbell-Sills et al., 2018) at T1 which was used to adjust for the severity of deployment-related stress in the models of post-deployment mental health outcomes. The DSS assessed exposure to potentially traumatic combat/deployment-related events (e.g. firing at the enemy/taking enemy fire, seeing severely wounded or dying people; theoretical range = 0–16). We therefore use the term deployment stress to reflect exposure to the assessed stressors encountered during deployment.

#### Sociodemographic and Army service variables

Sex, age, race/ethnicity (Non-Hispanic White, Non-Hispanic Black, Hispanic, or Other), marital status (currently married *v.* not), education (general equivalency, high school, or college/postgraduate degree), and Brigade Combat Team were included in PPDS models.

### Data analysis

We first examined separate prediction models, one for the five personality variables and two for the social network variable (one linear; one exploratory non-linear presented in the online Supplemental Materials), each adjusting for sociodemographic and service variables (described above), and lifetime history of the distress disorders or suicidal behaviors composite at baseline. Personality variables were standardized and treated as continuous; those significantly associated (*p* < 0.05) with distress disorders or suicidal behaviors were subsequently evaluated alongside the social network term to examine independent associations with the outcome. Finally, we added a neuroticism by social networks interaction term to test whether the hypothesized association between neuroticism and distress disorders or suicidal behaviors was moderated by social network size. We also explored personality by social network interaction terms for any other traits that were significant in the personality model. The base model predicting distress disorders or suicidal behaviors with main effects only was retained in cases wherein the personality by social network interaction term was not significant.

In the PPDS sample, survey-weights adjusted logistic regression models were used to evaluate the associations of pre-deployment (T0) personality and social network variables (and their interaction) with past 30-day distress disorders and suicidal behaviors at 3-months (T2) and 9-months (T3) after returning from deployment. Models adjusted for sociodemographic and service variables (T0), deployment stress (T1), and lifetime history of the relevant composite assessed at T0. See online Supplementary Material for a description of the NSS data analyses.

NSS and PPDS data are clustered and weighted; thus, the design-based Taylor series linearization method was used to estimate standard errors. Multivariable significance was examined using design-based Wald χ^2^ tests. Two-tailed *p* < 0.05 was considered significant. Effect sizes are described according to the proportion of mental health risk accounted for by every standard deviation change in the focal predictor (i.e. personality or social network variable). Because predictors of interest were standardized, the adjusted odds ratios can be directly compared to determine the relative strength of effects. All analyses were conducted using R Version 3.6.2 (R Core Team, 2021) with the following R libraries (survey, splines, gam, effects). The analyses were not pre-registered.

## Results

### Descriptive Analysis in PPDS sample

Personality traits were modestly related to social network scores in the PPDS sample (*r* = −0.20 to 0.24). See online Supplemental Table S1a for descriptive summaries, frequency distributions, and correlations among the personality and social network variables.

### Prospective analysis in PPDS sample

#### Distress disorders

The proportion of PPDS respondents meeting the criteria for the past 30-day distress disorders composite was 11.9% at T2 and 14.6% at T3. Adjusting for sociodemographic and service variables, deployment stress, and lifetime pre-deployment distress disorders, higher pre-deployment neuroticism and low conscientiousness independently (when considered together in the personality variable model) predicted the past 30-day distress disorders composite at both 3-months (T2) and 9-months (T3) after returning from deployment [Neuroticism: *3-months*, AOR = 1.40; 95% CI 1.23–1.60; χ^2^(1) = 25.44, *p* < 0.0005; *9-months*, AOR = 1.19; 95% CI 1.05–1.35; χ^2^(1) = 6.99, *p* = 0.008; Low Conscientiousness: *3-months*, AOR = 1.13; 95% CI 1.02–1.26; χ^2^(1) = 5.07, *p* = 0.024; *9-months*, AOR = 1.11; 95% CI 1.01–1.21; χ^2^(1) = 4.57, *p* = 0.033]. See online Supplemental Table S4a. Pre-deployment social network size significantly predicted the past 30-day distress disorders composite at T2 [linear term: AOR = 0.84; 95% CI 0.75–0.94; χ^2^(1) = 8.86, *p* = 0.003] and T3 [linear term: AOR = 0.85; 95% CI 0.77–0.94; χ^2^(1) = 10.18, *p* = 0.001].

The multivariable model at T2 revealed significant associations between neuroticism [AOR = 1.35; 95% CI 1.21–1.51; χ^2^(1) = 28.11, *p* < 0.0005] and past 30-day distress disorders at 3 months post-deployment. Thus, for each standard deviation increase in neuroticism there was a 35% increase in distress disorder risk 3 months after returning from deployment, adjusting for other variables. Low conscientiousness [AOR = 1.10; 95% CI 0.99–1.22; χ^2^(1) = 3.08, *p* = 0.079] and social networks [linear term; AOR = 0.90; 95% CI 0.80–1.02; χ^2^(1) = 2.75, *p* = 0.097] were no longer significant predictors. The interaction of personality and social networks was not significant at 3-months post-deployment for neuroticism [AOR = 0.97; 95% CI 0.88–1.07; χ^2^(1) = 0.37, *p* = 0.54] nor conscientiousness [AOR = 1.01; 95% CI 0.93–1.11; χ^2^(1) = 0.06, *p* = 0.80]. We therefore retained the main effects model for interpretation. Table 1 reports detailed results of the final prediction model for T2 outcomes.

**Table 1.** Final prediction model in the PPDS sample reporting associations of pre-deployment personality and social network variables with past-30-day distress disorders (a) or suicidal behavior (b) at *3-months post-deployment* (*N* = 4645), adjusting for sociodemographic factors, Brigade Combat Team (not shown here), deployment stress scale, and pre-deployment lifetime history of distress disorders or suicidal behaviors

	3-months Post-deployment					
	30-day distress disorders *Main effects model*			30-day Suicidal behaviors *Main effects model*		
	AOR (95% CI)	χ^2^	*p*	AOR (95% CI)	χ^2^	*p*
Age (years)	1.01 (0.98–1.04)	0.46	0.41	0.98 (0.93–1.02)	1.27	0.26
Female sex (reference: male)	1.37 (0.88–2.15)	1.94	0.16	1.97 (1.09–3.57)	5.00	0.025
Race/ethnicity (reference: White, non-Hispanic)		8.37	0.039		0.54	0.91
Black, non-Hispanic	1.25 (0.90–1.74)			0.77 (0.36–1.67)		
Hispanic	1.26 (0.94–1.68)			1.01 (0.59–1.71)		
Other, non-Hispanic	1.51 (1.03–2.22)			0.90 (0.34–2.39)		
Education (reference: high school degree)		6.33	0.042		0.98	0.61
General equivalency diploma	1.27 (0.85–1.89)			0.74 (0.30–1.79)		
College degree	0.74 (0.56–0.97)			1.10 (0.74–1.63)		
Marital status (reference: married)		1.33	0.51		6.39	0.041
Never married	0.85 (0.63–1.14)			1.49 (0.99–2.26)		
Other	1.03 (0.71–1.49)			1.94 (1.07–3.52)		
Lifetime (pre-deployment) distress disorders or suicidal behaviors	4.04 (3.25–5.02)	157.55	<0.0005	7.26 (4.77–11.03)	86.01	<0.0005
Combat/deployment stress	1.99 (1.77–2.25)	130.03	<0.0005	1.41 (1.21–1.64)	19.16	<0.0005
Neuroticism	1.35 (1.21–1.51)	28.11	<0.0005	1.32 (1.12–1.56)	10.80	0.001
Low conscientiousness	1.10 (0.99–1.22)	3.08	0.079			
Social networks (linear term)	0.90 (0.80–1.02)	2.75	0.097	0.84 (0.69–1.02)	3.14	0.076

The multivariable model at T3 revealed significant associations between neuroticism [AOR = 1.15; 95% CI 1.02–1.31; χ^2^(1) = 4.84, *p* = 0.028], social networks [linear term; AOR = 0.88; 95% CI 0.80–0.98; χ^2^(1) = 5.59, *p* = 0.018], and past 30-day distress disorders at 9 months post-deployment. Thus, for every standard deviation increase in neuroticism there was a 15% increase in distress disorders risk, adjusting for other variables, whereas each standard deviation increase in social network size was associated with a 12% reduction in risk at 9-months following return from deployment. Low conscientiousness was no longer a significant predictor [AOR = 1.09; 95% CI 0.99–1.20; χ^2^(1) = 3.13, *p* = 0.077]. The interaction of personality and social networks was not significant at 9-months post-deployment for neuroticism [AOR = 1.07; 95% CI 0.98–1.17; χ^2^(1) = 2.12, *p* = 0.15) nor conscientiousness [AOR = 1.05; 95% CI 0.96–1.15; χ^2^(1) = 1.05, *p* = 0.31]. We therefore retained the main effects model for interpretation. Table 2 reports detailed results of the final prediction model for T3 outcomes.

**Table 2.** Final prediction model in the PPDS sample reporting associations of pre-deployment personality and social network variables with past-30-day distress disorders (a) or suicidal behavior (b) at *9-months post-deployment* (*N* = 4645), adjusting for sociodemographic factors, Brigade Combat Team (not shown here), deployment stress scale, and pre-deployment lifetime history of distress disorders or suicidal behaviors

	9-months Post-deployment					
	30-day distress disorders *Main effects model*			30-day Suicidal behaviors *Neuroticism by social networks model*		
	AOR (95% CI)	χ^2^	*p*	AOR (95% CI)	χ^2^	*p*
Age (years)	1.01 (0.99–1.02)	0.69	0.41	1.01 (0.99–1.04)	1.01	0.31
Female sex (reference: male)	1.74 (1.17–2.60)	7.48	0.006	1.90 (1.10–3.27)	5.35	0.021
Race/ethnicity (reference: White, non-Hispanic)		3.53	0.32		1.93	0.59
Black, non-Hispanic	1.20 (0.84–1.73)			1.18 (0.73–1.91)		
Hispanic	1.14 (0.91–1.43)			1.20 (0.82–1.74)		
Other, non-Hispanic	1.26 (0.86–1.85)			1.30 (0.76–2.22)		
Education (reference: high school degree)		28.59	<0.0005		7.10	0.029
General equivalency diploma	1.74 (1.19–2.53)			1.62 (1.08–2.42)		
College degree	0.66 (0.53–0.81)			0.94 (0.66–1.32)		
Marital status (reference: married)		2.53	0.28		4.55	0.10
Never married	0.86 (0.69–1.07)			1.21 (0.83–1.76)		
Other	1.09 (0.83–1.44)			1.55 (1.00–2.42)		
Lifetime (pre-deployment) distress disorders or suicidal behaviors	3.19 (2.51–4.06)	88.73	<0.0005	3.98 (2.90–5.46)	73.52	<0.0005
Combat/deployment stress	1.72 (1.58–1.87)	153.10	<0.0005	1.27 (1.11–1.45)	11.79	0.001
Neuroticism	1.15 (1.02–1.31)	4.84	0.028	1.31 (1.13–1.52)	12.91	<0.0005
Low conscientiousness	1.09 (0.99–1.20)	3.13	0.077			
Social networks (linear term)	0.88 (0.80–0.98)	5.59	0.018	0.76 (0.66–0.87)	14.52	<0.0005
Neuroticism × Social networks (linear term)				1.12 (1.01–1.24)	4.58	0.032

#### Suicidal behaviors

The proportion of soldiers endorsing past 30-day suicidal behaviors was 2.9% at T2 and 5.7% at T3. In the personality trait model, only higher pre-deployment neuroticism predicted past 30-day suicidal behaviors at both 3-months (T2) and 9-months (T3) after returning from deployment [*3-months*, AOR = 1.31; 95% CI 1.07–1.62; χ^2^(1) = 6.47, *p* = 0.011; *9-months*, AOR = 1.24; 95% CI 1.03–1.49; χ^2^(1) = 5.14, *p* = 0.023]. See online Supplemental Table S4b. Pre-deployment social network size significantly predicted past 30-day suicidal behaviors at T2 [linear term: AOR = 0.79; 95% CI 0.65–0.95; χ^2^(1) = 6.17, *p* = 0.013] and T3 [linear term: AOR = 0.75; 95% CI 0.65–0.86; χ^2^(1) = 16.28, *p* < 0.0005].

The multivariable model at T2 revealed that higher neuroticism [AOR = 1.32; 95% CI 1.12–1.56; χ^2^(1) = 10.80, *p* = 0.001] predicted increased odds of past 30-day suicidal behaviors at 3 months post-deployment – accounting for 32% increased risk for every standard deviation increase in neuroticism scores, adjusting for other variables in the model. Social networks were not significantly associated with suicidal behaviors at 3-months post-deployment [linear term; AOR = 0.84; 95% CI 0.69–1.02; χ^2^(1) = 3.14, *p* = 0.076). The interaction of neuroticism by social networks was not significant at 3-months post-deployment [AOR = 1.05; 95% CI 0.93–1.17; χ^2^(1) = 0.61, *p* = 0.44]. We therefore retained the main effects model for interpretation. Table 1 reports detailed results of the final prediction model for T2 outcomes.

The multivariable model at T3 revealed a significant interaction effect between neuroticism and social networks [χ^2^(4) = 4.58, *p* = 0.032] on past 30-day suicidal behaviors at 9-months post-deployment. Visualization of the interaction revealed that neuroticism explained the increasing variance in suicidal behaviors at larger social networks. In contrast, the effect of neuroticism on suicidal behaviors was not strong in soldiers with the smallest social networks (i.e. 2 standard deviations below the mean) who, as a group, displayed an increased risk of suicidal behavior (i.e. above the sample average). See online Supplemental Fig. S4. Table 2 reports detailed results of the final prediction model for T3 outcomes.

### Supplementary analysis in NSS sample

**Fig. 1.**
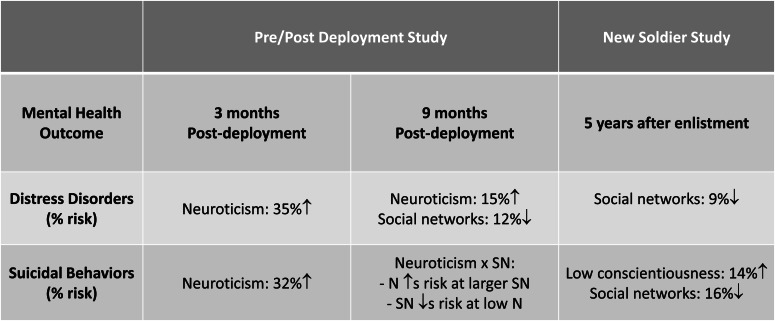
Summary of findings from the main multivariable models. Shown are significant (*p* < .05) predictors from the final model assessed before deployment (PPDS) or during basic training (NSS). Percent risk refers to the proportion of mental health risk accounted for by every standard deviation change in the focal predictor. Results of supplementary models investigating non-linear effects of social network size are not shown. N = neuroticism. SN = social networks.

Full results are presented in the online Supplemental Materials. Larger social networks reported during basic training predicted significantly reduced risk of distress disorders and suicidal behavior 5 years later – accounting for 9% and 16% reduction in risk, respectively, per standard deviation increase in social network size. Low conscientiousness was the only significant personality predictor of suicidal behavior, accounting for 14% increased risk per standard deviation increase, beyond social networks and other variables; it did not significantly interact with social network size. No personality traits significantly predicted distress disorders at 5-year follow-up. See Figure 1 for a summary of findings from the main multivariable models for the PPDS and NSS samples.

### Exploratory non-linear social network models

Modeling social networks as a non-linear term resulted in an improved model fit for only the distress disorders outcome measured 3 months following deployment (T2). All other models favored the linear social network term (see online Supplemental Materials for full results). Multivariable models at T2 revealed significant interactions between the non-linear social network term and both neuroticism and low conscientiousness. Neuroticism was associated with an increased risk of distress disorders at all but the highest social network scores (online Supplemental Fig. S2), whereas low conscientiousness predicted greater odds of distress disorders for soldiers with average social networks only (online Supplemental Fig. S3).

## Discussion

We examined prospective associations of personality traits and social network size with post-deployment distress disorders and suicidal behaviors in US Army soldiers. Pre-deployment neuroticism was consistently associated with increased post-deployment mental health risk, displaying associations with both distress disorders and suicidal behaviors at 3 and 9 months after return from deployment. Larger perceived social networks were associated with reduced risk of distress disorders and suicidal behaviors at 9 months post-deployment, accounting for unique variance beyond personality, socio-demographic and Army career characteristics (e.g. deployment history)^†^^1^. The hypothesis that neuroticism would interact with social network size was partially supported such that the positive association between neuroticism and suicidal behaviors at 9-months post-deployment depended on the size of soldiers' social networks. A supplementary analysis of data from an independent cohort further revealed that perceived social network size at the outset of basic training was significantly associated with the risk of distress disorders and suicidal behaviors approximately five years later. Considered together, these findings suggest that social network size contributes uniquely to predicting the incidence of distress disorders and suicidal behaviors beyond soldier characteristics including personality and lifetime history of these problems. The identification of a factor that is generally protective against distress disorders and suicidal behaviors – beyond the effects of other known risk factors (e.g. high neuroticism) – may help identify new avenues for reducing suicide and related mental health problems in soldiers.

The current data extend prior cross-sectional findings in military personnel post-deployment (Caska & Renshaw, 2013; James et al., 2013), and are the first, to our knowledge, to demonstrate a prospective link between neuroticism and mental health outcomes in service members. Because stressor exposure is common throughout all phases of the military lifecycle, especially during deployment, the exaggerated cognitive, affective and behavioral reactivity to stressors associated with high neuroticism may increase susceptibility to psychopathology in this population. Although the current findings cannot speak to the mechanisms that accounted for the prospective relationship between neuroticism and adverse mental health outcomes, they can help identify vulnerable soldiers preparing for deployment who may benefit from neuroticism-targeted interventions (Barlow et al., 2014; Sauer-Zavala et al., 2021). The lack of unique association between neuroticism measured in new recruits and distress disorders or suicidal behavior measured 5 years later may reflect changes in personality occurring throughout young adulthood (Roberts, Walton, & Viechtbauer, 2006) and during a time of multiple life transitions (e.g. adulthood, military life). Variability in unmeasured stressor exposure across soldiers throughout the 5-year period may also account for the observed findings.

Prior research indicates reliable, albeit lower magnitude (cf. neuroticism) cross-sectional associations between conscientiousness and anxiety and depressive disorders (Kotov et al., 2010). In this study, the prospective effects of low conscientiousness were modest in size and not statistically significant in multivariable models, except within the supplementary model of suicidal behavior in the new recruit cohort. Low conscientiousness may confer increased vulnerability to suicidal behaviors through ineffective emotion regulation, coping, or problem-solving (Carver & Connor-Smith, 2010; Javaras et al., 2012). The lack of significant associations of agreeableness and openness to experience with emotional disorders in our samples is consistent with prior cross-sectional research (Kotov et al., 2010; Watson & Naragon-Gainey, 2014). Extraversion is most reliably associated with a social anxiety disorder (Watson & Naragon-Gainey, 2014), which was not assessed across STARRS surveys. Depression is only modestly associated with extraversion at the broad trait level (Kotov et al., 2010) and tends to correlate mainly with the low positive emotionality facet (Naragon-Gainey, Watson, & Markon, 2009); however, items comprising the extraversion scale in the current study primarily assessed the sociability facet. The small number of items assessing each personality dimension offered limited coverage of the broad trait domains; it also likely accounts for the low internal consistency of some scales. Research is therefore needed to determine whether greater precision and explanatory power can be achieved with more reliable measures or by examining lower-level facets of the personality hierarchy (see for example Naragon-Gainey et al., 2009).

The social environment is also linked to mental health functioning, both cross-sectionally and prospectively (Holma et al., 2010; Saris et al., 2017; Teo et al., 2013). Unit cohesion – a unique form of social network quality within the military – has been shown to protect against mental disorders and suicidal behavior (Anderson et al., 2019; Campbell-Sills et al., 2022). Here, we examined social networks more broadly – endeavoring to increase the generalizability of our findings beyond military samples. The current study demonstrated that service members reporting smaller social networks were at greater *future* risk for meeting criteria for a distress disorder or suicidal behavior following their return from deployment, or at a naturalistic follow-up assessment approximately 5 years following enlistment. Results largely supported a linear relationship between social network size and mental health outcomes; the only exception was soon after soldiers returned from deployment (discussed below). Our findings dovetail with recent observations that resilient mental health trajectories in the first several years of military service – i.e., those characterized by stable, low psychological distress or posttraumatic stress symptoms – are predicted by increased social support from family, friends, and military peers and leaders (Dell et al., 2022). Identifying new recruits or soldiers preparing for deployment who report particularly impoverished social networks could allow for early intervention targeting mechanisms hypothesized to underpin social disconnection. For example, interventions that enhance social group membership (Haslam, Cruwys, Haslam, Dingle, & Chang, 2016), positive emotions and approach behaviors (Taylor, Pearlstein, Kakaria, Lyubomirsky, & Stein, 2020), and/or reduce malapative social cognition (e.g. cognitive behavioral approaches; Cacioppo *et al*. 2015a, 2015b) have been shown to improve social connectedness and may therefore be able to alter mental health trajectories of soldiers.

This study is the first, to our knowledge, to evaluate whether social network size influences the link between personality and psychopathology—finding modest support for the hypothesis that larger social networks would buffer the effects of neuroticism on mental health. A non-linear buffering effect was observed wherein the risk of distress disorders was *not* elevated for soldiers with high neuroticism who reported very large social networks (i.e. 2 s.d. above mean social network size). This buffering effect was observed 3 months after return from deployment – a challenging time of transition when the availability of many social contacts on which to rely for support may be particularly beneficial, especially for soldiers prone to elevated stressor reactivity. A different pattern of findings emerged for suicidal behavior 9-months post-deployment, wherein neuroticism was not related to the risk of suicidal behaviors in soldiers with the smallest social networks but predicted increasing risk as social network size increased. This may suggest that even individuals lower in neuroticism are at increased risk of suicidal behaviors if the social network is especially impoverished. An exploratory analysis also revealed that social network size moderated the relationship between conscientiousness and risk for distress disorders at 3 months post-deployment, suggesting perceived availability of social contacts may influence maladaptive processes associated with low conscientiousness (e.g. ineffective coping or emotion dysregulation; Carver & Connor-Smith, 2010; Javaras *et al*. 2012). Overall, our findings underscore the need to consider interactions between personality and social environment factors in predicting psychopathology – though the precise nature and meaning of those interactions remain to be determined.

Interpretation of the current findings should be considered alongside study limitations. Personality and social network size were assessed via self-report. Replication using informant reports and measures that assess other indicators of social functioning (e.g. objective measures of network size, interconnectedness, and frequency of social activities; Saris et al., 2017) would buttress the current findings. Although brief egocentric social network surveys like the one used in this study have practical advantages (e.g. efficient mass administration), they are limited in measuring the precise nature (e.g. family, friends) and function (e.g. engagement in leisure activities, availability of emotional support) of different relationships within one's network, as well as structural features of the network (e.g. cohesion). Thus, the ability to evaluate non-linear associations between network size and mental health outcomes may have been obscured by not capturing specific aspects of one's social connections and their functions (Falci & McNeely, 2009). Egocentric surveys also do not capture dependencies among individuals within one's network (e.g. Schaefer, Kornienko, and Fox, 2011), including mutual influences of mental health across members of the network (Rosenquist, Fowler, & Christakis, 2011). Such dependencies may alter the strength or direction of associations between social networks and mental health, including possible positive associations among those with psychopathology (e.g. contagion effects), suggesting the current findings may underestimate the impact of social networks on mental health. Both studies relied on self-reported mental health outcomes; replication using clinician-administered diagnoses is needed. Although examining prospective associations between our hypothesized predictors and subsequent mental health outcomes when including stringent baseline controls is an advance over prior cross-sectional investigations, this was an observational study and therefore causality cannot be inferred. Social networks are dynamic throughout the life course (Small, Pamphile, & McMahan, 2015) and would be expected to change over the military lifecycle. Such changes may vary depending on the nature of the relationship (e.g. civilian *v.* military connections) and would attenuate the relationship between baseline social networks and future mental health outcomes, suggesting our results likely provide a conservative estimate of concurrent or more proximal associations between social networks and psychopathology. Future research should measure changes in social networks and personality occurring alongside changes in psychopathology so that prospective and concurrent associations can be modeled. Replication of findings in civilian samples who are exposed to high levels of stressors based on job-related (e.g. emergency service personnel) or other factors (e.g. poverty, discrimination) is also needed.

## Conclusion

Elevated neuroticism and having few social connections uniquely predicted increased risk for distress disorders and suicidal behavior among U.S. Army soldiers returning from deployment. Small social networks also predicted these mental health outcomes 5 years following enlistment. These observations occurred when including stringent baseline controls, suggesting both factors are important to assess and potentially target in the prevention and early intervention programs in military settings. There may be times when social network size influences the effects of neuroticism on mental health, though further research is needed to confirm whether and under what conditions such effects occur. Extending the current findings to civilian populations at risk of high stressor exposure is now needed to further their public health reach.

## Footnotes

† The note appear after the main text.

## Note

1 Interpretation of socio-demographic and Army career variables in the prediction models is beyond the scope of this paper. Interested readers may consult the following paper for more details (Zuromski et al., 2020).

## Supplementary material

For supplementary material accompanying this paper visit https://doi.org/10.1017/S0033291722002082. click here to view supplementary material
