# Supplementary material for: Social network size and personality traits independently and prospectively predict distress disorders and suicidal behavior in U.S. Army soldiers
Source: Psychol Med. 2022 Aug 18;53(11):5081–90. doi: 10.1017/S0033291722002082 (PMC9938080; doi:10.1017/S0033291722002082)

**Supplemental Materials**

**Supplemental Methods**

**Participants**

Data were obtained from two Army STARRS cohorts comprised of representative samples of Army soldiers (for detailed descriptions of study design and procedures see Heeringa et al., 2013; Kessler, Colpe, et al., 2013; Ursano et al., 2014). Human Subjects Committees of the collaborating institutions approved recruitment, consent, and data protection procedures. Soldiers provided written informed consent for survey participation. Consent to link responses to their Army/Department of Defense (DoD) administrative records was requested separately.

*Pre/Post Deployment Study (PPDS):* Regular Army soldiers from three Brigade Combat Teams (BCTs) were recruited to take part in a longitudinal multi-wave panel survey before deploying to Afghanistan in 2012. The baseline evaluation occurred 1-2 months prior to deployment (T0). Follow-up assessments occurred within 1 month of their return to the U.S. (T1), 3 months later (T2), and 9 months later (T3). Of the 9,949 soldiers who presented for duty in the BCTs at T0, 95.3% consented to the survey, and 86.0% completed the survey and consented to administrative record linkage. Most of these soldiers subsequently deployed to Afghanistan (*n*=7,742; 90.5%). Prediction models were tested within soldiers who completed surveys at all waves (*n*=4,645; 60.0% of the eligible sample of deployed soldiers). Response propensity and post-stratification weighting factors were developed and applied in all PPDS analyses (Heeringa, West, & Berglund, 2010). The weights included (1) a propensity-based adjustment for baseline attrition due to incomplete surveys and inability to link to Army/DoD administrative data; (2) post-stratification of the propensity weights to sociodemographic and military career characteristics of all soldiers in the three combined Brigade Combat Teams that deployed to Afghanistan after T0; and (3) a propensity-based attrition adjustment to account for loss of participants due to incomplete data in one or more of the follow-up waves (for further details see Kessler, Heeringa et al., 2013). The PPDS sample demographic composition was as follows: age (M =26.27, GMD = 6.57), sex (94% male, 6% female), race (65.3% non-Hispanic White, 10.1% non-Hispanic Black, 16.4% Hispanic, 8.3% non-Hispanic other race); education (72.4% high school, 6.3% GED, 21.4% college); marital status (36.5% never married, 55.4% currently married, 8.1% other).

*New Soldier Study (NSS):* Soldiers (including Regular Army, Guard, and Reserve) were first surveyed shortly before Basic Combat Training at three Army installations from April 2011-November 2012. The majority of selected soldiers consented to the survey (99.9%), completed the survey (93.5%), and consented to linkage of their survey responses to their Army/DoD administrative records (77.1% of survey completers; 38,507 soldiers; for a description of the sample see Rosellini et al., 2015). The current prospective analysis included 6,216 soldiers from that sample (49% Regular Army, 33% Guard, 18% Reserve), whose NSS survey data was successfully linked to follow-up surveys completed approximately 5 years later as part of the STARRS Longitudinal Study (STARRS-LS; time interval in years from baseline to follow-up, M=5.25, range=3.85 to 6.83).

LS recruitment was based on a complex multi-stage nonproportional sampling scheme. The initial LS sampling frame divided baseline Army STARRS survey participants into three strata for differential sampling to maximize statistical power for fixed data collection costs that would not allow follow-up of all baseline respondents. Stratum 1 (n=22,176) included respondents who reported having a lifetime history of suicidality and/or any clinically significant mental disorder, or were ever in the Special Forces. Stratum 2 (n=26,833) included additional respondents (i.e., not in Stratum 1) who were either female or in the Army Reserve or Army National Guard. Stratum 3 (n=23,378) included all remaining baseline survey respondents. The first STARRS-LS survey (LS1; September 2016-April 2018) attempted to administer a self-report follow-up survey to 100% of Stratum 1 and probability samples of 67% of Stratum 2 and 50% of Stratum 3. The final LS Wave 1 survey data were weighted to adjust for the differential sampling. The current prospective analysis included 6,216 soldiers from the New Soldier Study sample described in Rosellini et al. (2015), whose baseline data were successfully linked to STARRS-LS wave 1 survey data. The sample demographic composition was as follows: age (M =21.68, GMD = 4.21), sex (78.5% male, 21.5% female), race (62.3% non-Hispanic White, 14.6% non-Hispanic Black, 14.9% Hispanic, 8.2% non-Hispanic other race); education (72.9% high school, 14.9% GED, 12.2% college); marital status (85.4% not currently married, 14.6% currently married). Twenty-four percent of soldiers were deployed during the follow-up period; 76% were never deployed.

**Measures**

**Personality**. The PPDS and NSS baseline surveys contained a pool of 87 questions that were adapted from previously validated self-report personality inventories (see Rosellini et al., 2017) and were intended to assess 24 personality traits of interest. We focus this investigation on the five higher-order dimensions of neuroticism (7 items: e.g., “I have a harder time than most people handling stressful situations”; “I worry about things a lot more than other people”; PPDS T0 α =.82, NSS α =.76), agreeableness (3 items: e.g., “I feel good when I help people”; “I am a very modest person, the sort of person who never brags about my accomplishments”; PPDS T0 α=.54, NSS α=.48), openness to experience (4 items: e.g., “I am pretty set in my ways”; “I am open-minded about how other people live their lives”; PPDS T0 α=.37, NSS α=.34), extraversion (3 items: e.g., “I am much more shy than most people”; “I am pretty quiet around people I don’t know well”; PPDS T0 α=.68, NSS α=.68), and low conscientiousness (2 items: “I sometimes don’t follow through on things I promise to do”; “I am often disorganized”; PPDS T0 α=.54, NSS α=.41). Each scale was determined to be unidimensional (all item factor loadings ≥ .40) through exploratory and confirmatory factor analyses conducted in the NSS, PPDS, and combined samples. The scale was prefaced by, “The next questions are about how you see yourself. How well does each of the following statements describe you?”. Items were rated on a 5-point Likert-style scale with anchors of ‘*not at all like me’* to ‘*exactly like me*’. Ratings were coded 0-4 and summed to create a total score for each trait. Standardized scores were used in the prediction models.

**Social networks.** The social network scale comprised 4 items that assessed the size of different aspects of Soldiers’ affiliative networks. Items were prefaced with, “How many people do you have in your personal life of the following sorts?” and were rated on a 10-point scale referencing 0, 1, 2, 3, 4, 5, 6-10, 11-20, 21-30, or 31 or more people. Ratings were coded 0-9. Items referenced: “people you do things with, like watch TV together, go out for a drink or movie together, or play cards”; “people who you feel really close to”; “people who really care for you and would be there if you needed them”; and “family or friends who need you and rely on you for help when they need it.” Exploratory factor analysis revealed that all items loaded strongly on one factor (item-factor loadings: PPDS=.66-.89, NSS=.61-.86). Thus, items were summed to create a total social network score with higher scores reflecting stronger social networks (scale range = 0-36). Internal consistency was good in both the PPDS T0 (α=.85) and NSS (α=.81) samples.

**Mental disorders and suicidal behaviors**. Mental disorders were assessed in the surveys using items adapted from the Composite International Diagnostic Interview Screening Scales (CIDI-SC; (Kessler & Ustun, 2004)), the civilian PTSD Checklist for DSM-IV (Weathers, Litz, Herman, Huska, & Keane, 1993), and the PTSD Checklist for DSM-5 (Blevins, Weathers, Davis, Witte, & Domino, 2015). Diagnoses based on the survey items demonstrated satisfactory concordance with independent clinical diagnoses (Kessler, Santiago, et al., 2013). An expanded self-report version of the Columbia Suicide Severity Rating Scale (Posner et al., 2011) was used to assess suicidal behaviors, defined as reporting any suicide ideation, plan, or attempt. The primary outcomes in both the PPDS and NSS samples were composite indices reflecting presence vs. absence of (1) any distress disorder (i.e., any diagnosis of posttraumatic stress disorder [PTSD], major depressive episode [MDE], generalized anxiety disorder [GAD]) and (2) suicidal behavior (i.e., any suicide ideation, plan, or attempt) in the past 30 days (PPDS) or past 12 months (NSS/STARRS-LS). Presence vs. absence of any lifetime distress disorder or suicidal behavior at baseline (i.e., at the time of the PPDS T0 or NSS survey) was included as a covariate in the predictive models.

**Sociodemographic and Army service variables**. Sex, age, race/ethnicity (Non-Hispanic White, Non-Hispanic Black, Hispanic, or Other), marital status (currently married vs. not), and education (general equivalency, high school, or college/postgraduate degree) were included in both PPDS and NSS models. PPDS analyses also adjusted for BCT. NSS analyses also adjusted for site of basic training, service component (Regular Army, Guard, or Reserve), and deployment history (yes/no; 24% endorsed having been deployed one or more times).

**Data Analysis for NSS sample**

Survey-weights adjusted logistic regression models were used to evaluate the associations of baseline personality and social network variables (and their interaction) with past 12-month distress disorder and suicidal behavior outcomes measured during the STARRS-LS wave 1 survey. Models adjusted for sociodemographic and service variables, and lifetime history of the distress disorders or suicidal behaviors composite at baseline.

**Supplemental Results**

**Descriptive Analysis – NSS sample**

Personality traits were modestly related to social network scores (*r* range = -.12 to .18, NSS sample). See Supplemental Table 1b for descriptive summaries, frequency distributions, and correlations among the personality and social network variables.

**Prospective Analysis – NSS sample**

**NSS sample – Distress Disorders:** The proportion of soldiers meeting criteria for the past 12-month distress disorders composite at follow-up was 29.6%. In the personality model, none of the individual traits measured at baseline significantly predicted past 12-month distress disorders. See Supplemental Table 2. Baseline social network size modeled as a linear term (AOR=0.91; 95% CI=0.84-0.99; *Χ^2^*(1)=4.85, *p*=.028) significantly predicted past 12-month distress disorders. The LRT did not support significantly improved fit with inclusion of the non-linear term (*p*=.06). Thus, adjusting for demographic characteristics, deployment history, and (pre-enlistment) lifetime history of a distress disorder diagnosis, every standard deviation increase in social network size measured during basic training accounted for 9% reduced risk of receiving a distress disorders diagnosis approximately 5 years later. See Supplemental Table 3.

**NSS sample – Suicidal behaviors:** 15.6% of soldiers endorsed past 12-month suicidal behaviors at follow-up. In the personality model, extraversion and low conscientiousness at baseline predicted past 12-month suicidal behaviors (Extraversion: AOR=0.89; 95% CI=0.80-0.99; *Χ^2^*(1)=5.00, *p*=.025; Low Conscientiousness: AOR=1.11; 95% CI=1.01-1.22; *Χ^2^*(1)=4.29, *p*=.038). See Supplemental Table 2. Baseline social network size modeled as a linear term (AOR=0.82; 95% CI=0.76-0.90; *Χ^2^*(1)=20.14, *p*<.0005) significantly predicted past 12-month suicidal behavior. The LRT did not support improved fit with inclusion of the non-linear term (*p*=.60). We therefore examined extraversion, low conscientiousness, and the linear social network term as predictors in a subsequent model. See Supplemental Table 3 for full model results. Results revealed that low conscientiousness (AOR=1.14; 95% CI=1.05-1.24; *Χ^2^*(1)=8.87, p=.003) and social network size (AOR=0.84; 95% CI=0.77-0.92; *Χ^2^*(1)=15.09, p<.0005) predicted past 12-month suicidal behavior. Extraversion was not a significant predictor (AOR=0.92; 95% CI=0.84-1.01; *Χ^2^*(1)=3.40, p=.065), nor were the personality by social networks interaction terms (Low Conscientiousness x Social Network Size: AOR=1.03; 95% CI=0.95-1.11; *Χ^2^*(1)=0.41, p=.52; Extraversion x Social Network Size: AOR=0.99; 95% CI=0.91-1.07; *Χ^2^*(1)=0.14, p=.71). Thus, adjusting for other factors, for each standard deviation increase in social network size during basic training there was a 16% reduction in risk of suicidal behaviors approximately 5 years later, and a 14% increase in risk of suicidal behaviors for every standard deviation increase in low conscientiousness.

**PPDS sample – Exploratory Non-Linear Social Network Models**

Separate models evaluated the linear (presented in the main text) and non-linear associations between the standardized social network score and the outcomes. Non-linear relationships were modeled using a natural cubic spline with four degrees of freedom. A likelihood ratio test (LRT) was conducted to compare the model using natural cubic spline terms (i.e., a piecewise third-degree polynomial) and the model with a linear term.

**PPDS sample – Distress Disorders:** Pre-deployment social network size modeled as a non-linear term significantly predicted the past 30-day distress disorders composite at T2 (natural cubic spline terms: *Χ^2^*(4)=49.21, *p*<.0005; LRT *p*=.013) and T3 (natural cubic spline terms: *Χ^2^*(4)=24.75, *p*<.0005, LRT *p*=.28). The LRT suggested the non-linear social network term resulted in improved model fit at T2 but not T3. We therefore conducted an exploratory multivariable model at T2 including the non-linear social network term and personality variables that were significant predictors from the personality only model (i.e., neuroticism and low conscientiousness). This model revealed a significant interaction effect between neuroticism and social networks (*Χ^2^*(4)=11.62, *p*=.02) on past 30-day distress disorders at 3-months post-deployment. Visualization of the interaction revealed that neuroticism predicted higher incidence of the distress disorders composite at all but the highest social network values (i.e., 2 standard deviations above the mean). See Supplemental Figure 2. For soldiers with the largest social networks, neuroticism was not associated with increased risk of distress disorders. An exploratory low conscientiousness by non-linear social networks interaction analysis revealed a significant interaction effect on the outcome at 3-months post-deployment (*Χ^2^*(4)=18.89, *p*=.001). Lower conscientiousness was associated with greater odds of the distress disorders composite for soldiers with average social networks, but was not associated with elevated risk for soldiers with below or above average networks. See Supplemental Figure 3. Supplemental Table 5 reports detailed results of the final non-linear prediction model for T2 outcomes.

**PPDS sample – Suicidal behaviors:** Pre-deployment social network size modeled as a non-linear term significantly predicted past 30-day suicidal behaviors at T2 (natural cubic spline terms: *Χ^2^*(4)=10.49, *p*=.033; LRT *p*=.62) and T3 (natural cubic spline terms: *Χ^2^*(4)=26.50, *p*<.0005, LRT *p*=.61); however, the non-significant LRT supported interpreting the linear social network term at both T2 and T3.

Supplemental Table 1a

Descriptive summaries and zero-order correlations for the personality and social network variables in the Pre-Post Deployment Study (PPDS).

| Variable | Mean (SD) | Range | 1 | 2 | 3 | 4 | 5 | 6 |
| --- | --- | --- | --- | --- | --- | --- | --- | --- |
| 1. Social Networks | 16.53 (7.45) | 0 - 36 | 1.00 |  |  |  |  |  |
| 1. Neuroticism | 5.60 (4.68) | 0 - 28 | -.20 | 1.00 |  |  |  |  |
| 1. Agreeableness | 7.36 (2.45) | 0 - 12 | .16 | .18 | 1.00 |  |  |  |
| 1. Openness | 9.42 (2.52) | 0 - 16 | .16 | -.22 | .06 | 1.00 |  |  |
| 1. Extraversion | 7.61 (2.83) | 0 - 12 | .24 | -.45 | -.04 | .33 | 1.00 |  |
| 1. Low Conscientiousness | 1.35 (1.50) | 0 - 8 | -.17 | .52 | .03 | -.09 | -.26 | 1.00 |

Supplemental Table 1b

Descriptive summaries and zero-order correlations for the personality and social network variables in the New Soldier Study (NSS).

| Variable | Mean (SD) | Range | 1 | 2 | 3 | 4 | 5 | 6 |
| --- | --- | --- | --- | --- | --- | --- | --- | --- |
| 1. Social Networks | 19.17 (7.10) | 0 - 36 | 1.00 |  |  |  |  |  |
| 1. Neuroticism | 7.66 (5.01) | 0 - 28 | -.12 | 1.00 |  |  |  |  |
| 1. Agreeableness | 7.90 (2.35) | 0 - 12 | .10 | .16 | 1.00 |  |  |  |
| 1. Openness | 9.90 (2.28) | 0 - 16 | .08 | -.27 | .02 | 1.00 |  |  |
| 1. Extraversion | 7.08 (2.85) | 0 - 12 | .18 | -.40 | -.08 | .33 | 1.00 |  |
| 1. Low Conscientiousness | 1.86 (1.67) | 0 - 8 | -.08 | .47 | .04 | -.13 | -.22 | 1.00 |

Supplemental Table 2

Prediction model in the NSS sample reporting associations of five-factor personality traits measured during basic combat training with past 12-month distress disorders (A) or suicidal behavior (B) measured approximately 5 years later (N=6,216), adjusting for (not shown here) sociodemographic factors, site of Basic Combat Training, Army service component (Regular, Guard, or Reserve), deployment history, and lifetime history of distress disorders or suicidal behaviors at baseline.

|  | (A) 12-month Distress Disorders | | | (B) 12-month Suicidal Behaviors | | |
| --- | --- | --- | --- | --- | --- | --- |
|  | AOR (95% CI) | *Χ^2^* | *p* | AOR (95% CI) | *Χ^2^* | *p* |
| Neuroticism | 1.08 (0.96-1.23) | 1.57 | .21 | 1.09 (0.98-1.21) | 2.65 | .10 |
| Agreeableness | 1.05 (0.97-1.15) | 1.47 | .23 | 1.01 (0.91-1.10) | 0.01 | .92 |
| Openness | 1.07 (0.96-1.18) | 1.46 | .23 | 1.08 (0.97-1.20) | 1.84 | .18 |
| Extraversion | 0.98 (0.88-1.09) | 0.16 | .69 | 0.89 (0.80-0.99) | 5.00 | .025 |
| Low conscientiousness | 1.07 (0.98-1.17) | 2.26 | .13 | 1.11 (1.01-1.22) | 4.29 | .038 |

*Note*. AOR=adjusted odds ratio; CI=confidence interval. Personality scores were standardized to facilitate interpretation of the AORs.

Supplemental Table 3

Final prediction model for the NSS sample reporting associations of personality and social network variables measured during basic combat training with past 12-month distress disorders (A) or suicidal behavior (B) measured approximately 5 years later (N=6,216), adjusting for sociodemographic factors, site of Basic Combat Training (not shown here), Army service component (Regular, Guard, or Reserve), deployment history, and lifetime history of distress disorders or suicidal behaviors at baseline.

|  | (A) 12-month Distress Disorders | | | (B) 12-month Suicidal Behaviors | | |
| --- | --- | --- | --- | --- | --- | --- |
|  | AOR (95% CI) | *Χ^2^* | *p* | AOR (95% CI) | *Χ^2^* | *p* |
| Age (years) | 1.01 (0.98-1.03) | 0.32 | .57 | 1.01 (0.98-1.04) | 0.41 | .52 |
| Female sex (reference: male) | 2.31 (1.91-2.81) | 72.66 | <.0005 | 1.45 (1.17-1.79) | 11.71 | .001 |
| Race/ethnicity (reference: White, non-Hispanic) |  | 4.63 | .20 |  | 2.49 | .48 |
| Black, non-Hispanic | 0.81 (0.65-1.00) |  |  | 0.78 (0.56-1.08) |  |  |
| Hispanic | 1.01 (0.79-1.28) |  |  | 1.00 (0.80-1.25) |  |  |
| Other, non-Hispanic | 1.16 (0.88-1.51) |  |  | 0.96 (0.69-1.35) |  |  |
| Service component (reference: Regular Army) |  | 31.99 | <.0005 |  | 9.69 | .008 |
| Guard | 0.62 (0.51-0.77) |  |  | 0.77 (0.62-0.95) |  |  |
| Reserve | 0.52 (0.40-0.68) |  |  | 0.72 (0.55-0.95) |  |  |
| Education (reference: high school degree) |  | 26.31 | <.0005 |  | 5.14 | .077 |
| General equivalency diploma | 0.968 (0.79-1.18) |  |  | 0.97 (0.77-1.24) |  |  |
| College degree | 0.43 (0.31-0.59) |  |  | 0.68 (0.49-0.95) |  |  |
| Married (reference: not married) | 1.05 (0.83-1.32) | 0.17 | .68 | 1.13 (0.84-1.51) | 0.66 | .42 |
| Lifetime (baseline) distress disorders (panel A) or suicidal behaviors (panel B) | ﻿6.06 (5.08-7.23) | 401.79 | <.0005 | ﻿2.68 (2.20-3.27) | 95.80 | <.0005 |
| Deployment history (reference: no) | 1.22 (1.01-1.47) | 4.10 | .043 | 1.00 (0.77-1.31) | 0.00 | 1.00 |
| **Extraversion*** |  |  |  | **0.92 (0.84-1.01)** | **3.40** | **.065** |
| **Low conscientiousness*** |  |  |  | **1.14 (1.05-1.24)** | **8.87** | **.003** |
| **Social networks (linear term)** | **.91 (.84-.99)** | **4.85** | **.028** | **0.84 (0.77-0.92)** | **15.09** | **<.0005** |

*Note*. Only personality traits that were significant (*p*<.05) in the personality variable model were included in the model with the social network variable. Personality and social network scores were standardized. The personality by social network interaction terms were not significant (*p*>.05); this table therefore reports results of the main effects model only. The bold values highlight the central predictor(s) evaluated with the final regression model. AOR=adjusted odds ratio; CI=confidence interval. *Not included in the distress disorders final model.

Supplemental Table 4a

Prediction model in the PPDS sample reporting associations of pre-deployment five-factor personality traits with post-deployment *distress disorders* (N=4,645), adjusting for (not shown here) sociodemographic factors, Brigade Combat Team, deployment stress scale, and pre-deployment lifetime history of distress disorders.

|  | 30-day Distress Disorders (PTSD, MDE, or GAD) | | | | | |
| --- | --- | --- | --- | --- | --- | --- |
|  | 3 months post-deployment | | | 9 months post-deployment | | |
|  | AOR (95% CI) | *Χ^2^* | *p* | AOR (95% CI) | *Χ^2^* | *p* |
| Neuroticism | 1.40 (1.23-1.60) | 25.44 | <.0005 | 1.19 (1.05-1.35) | 6.99 | .008 |
| Agreeableness | 1.03 (.91-1.17) | 0.21 | .64 | 1.00 (0.91-1.09) | 0.01 | .92 |
| Openness | 0.93 (0.81-1.08) | 0.83 | .36 | 0.99 (0.91-1.09) | 0.04 | .85 |
| Extraversion | 1.12 (0.97-1.28) | 2.48 | .12 | 1.04 (0.94-1.16) | 0.55 | .46 |
| Low conscientiousness | 1.13 (1.02-1.26) | 5.07 | .024 | 1.11 (1.01-1.21) | 4.57 | .033 |

*Note*. PTSD=posttraumatic stress disorder; MDE=major depressive episode; GAD=generalized anxiety disorder; AOR=adjusted odds ratio; CI=confidence interval. Personality scores were standardized to facilitate interpretation of the AORs.

Supplemental Table 4b

Prediction model in the PPDS sample reporting associations of pre-deployment five-factor personality traits with post-deployment *suicidal behavior* (N=4,645), adjusting for (not shown here) sociodemographic factors, Brigade Combat Team, deployment stress scale, and pre-deployment lifetime history of suicidal behavior.

|  | 30-day Suicidal Behaviors | | | | | |
| --- | --- | --- | --- | --- | --- | --- |
|  | 3 months post-deployment | | | 9 months post-deployment | | |
|  | AOR (95% CI) | *Χ^2^* | *p* | AOR (95% CI) | *Χ^2^* | *p* |
| Neuroticism | 1.31 (1.07-1.62) | 6.47 | .011 | 1.24 (1.03-1.49) | 5.14 | .023 |
| Agreeableness | 0.92 (0.78-1.09) | 0.88 | .35 | 0.97 (0.86-1.10) | 0.21 | .64 |
| Openness | 0.96 (0.79-1.17) | 0.15 | .70 | 0.95 (0.80-1.12) | 0.40 | .53 |
| Extraversion | 0.99 (0.78-1.26) | 0.00 | .95 | 1.04 (0.86-1.26) | 0.18 | .67 |
| Low conscientiousness | 1.08 (0.87-1.34) | 0.53 | .47 | 1.11 (0.93-1.32) | 1.29 | .26 |

*Note*. AOR=adjusted odds ratio; CI=confidence interval. Personality scores were standardized to facilitate interpretation of the AORs.

Supplemental Table 5

Final exploratory prediction models for the PPDS sample reporting associations of pre-deployment personality and *non-linear* social network variables with past-30-day distress disorders at *3-months post-deployment* (N=4,645), adjusting for sociodemographic factors, Brigade Combat Team (not shown here), deployment stress scale, and pre-deployment lifetime history of distress disorders.

|  | 30-day Distress Disorders (PTSD, MDE, or GAD) | | | | | |
| --- | --- | --- | --- | --- | --- | --- |
|  | 3 months post-deployment  *Neuroticism by social networks model* | | | 3 months post-deployment  *Low conscientiousness by social networks model* | | |
|  | AOR (95% CI) | *Χ^2^* | *p* | AOR (95% CI) | *Χ^2^* | *p* |
| Age (years) | 1.01 (0.98-1.04) | 0.43 | .51 | 1.01 (0.98-1.03) | 0.27 | .60 |
| Female sex (reference: male) | 1.40 (0.90-2.17) | 2.23 | .14 | 1.40 (0.91-2.16) | 2.37 | .12 |
| Race/ethnicity (reference: White, non-Hispanic) |  | 8.30 | .04 |  | 7.78 | .051 |
| Black, non-Hispanic | 1.27 (0.91-1.76) |  |  | 1.28 (0.92-1.78) |  |  |
| Hispanic | 1.26 (0.94-1.68) |  |  | 1.24 (0.93-1.65) |  |  |
| Other, non-Hispanic | 1.50 (1.02-2.19) |  |  | 1.47 (1.00-2.17) |  |  |
| Education (reference: high school degree) |  | 6.31 | .043 |  | 6.26 | .044 |
| General equivalency diploma | 1.27 (0.85-1.88) |  |  | 1.27 (0.86-1.87) |  |  |
| College degree | 0.74 (0.56-0.98) |  |  | 0.74 (0.56-0.98) |  |  |
| Marital status (reference: married) |  | 1.48 | .48 |  | 1.46 | .48 |
| Never married | 0.84 (0.62-1.13) |  |  | 0.83 (0.62-1.13) |  |  |
| Other | 1.03 (0.70-1.51) |  |  | 1.01 (0.69-1.47) |  |  |
| Lifetime (pre-deployment) distress disorders | ﻿4.07 (3.28-5.05) | 160.88 | <.0005 | ﻿4.06 (3.28-5.03) | 165.19 | <.0005 |
| Combat/deployment stress | 1.99 (1.76-2.24) | 127.44 | <.0005 | 1.98 (1.76-2.23) | 129.67 | <.0005 |
| Neuroticism | 1.17 (.80-1.71) | 0.67 | .41 | 1.35 (1.21-1.51) | 29.51 | <.0005 |
| Low conscientiousness | 1.10 (0.99-1.23) | 3.39 | .066 | 1.01 (0.68-1.50) | 0.00 | .97 |
| Social networks (natural cubic spline) |  | 27.87 | <.0005 |  | 45.17 | <.0005 |
| **Personality x Social networks (natural cubic spline)** |  | **11.62** | **.02** |  | **18.89** | **.001** |

*Note*. Only personality traits that were significant (p<.05) in the personality variable model were included in the model with the social network variable. Personality and social network scores were standardized. AORs are not interpretable for the natural cubic spline terms and therefore are not reported. The interaction between personality and social networks predicting the distress disorders composite 3-months post-deployment can be seen in Supplemental Figures 2 and 3. The bold values highlight the central predictor(s) evaluated with the final regression model. PTSD=posttraumatic stress disorder; MDE=major depressive episode; GAD=generalized anxiety disorder; AOR=adjusted odds ratio; CI=confidence interval.

Supplemental Figure 1a

Frequency distributions for the personality and social network variables in the New Solider Study.

Supplemental Figure 1b

Frequency distributions for the personality and social network variables in the Pre-Post Deployment Study.

Supplemental Figure 2a

Non-linear association between pre-deployment neuroticism and probability of past 30-day distress disorders in the PPDS sample measured 3 months following return from deployment (T2) plotted at different levels of pre-deployment social network scores. The shaded area surrounding the line represents the 95% confidence interval. See Supplemental Figure 2b for an alternate visualization of the interaction.


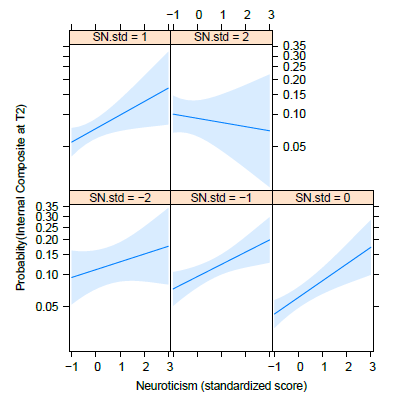


Supplemental Figure 2b

Non-linear association between perceived social networks measured pre-deployment and probability of past 30-day distress disorders in the PPDS sample measured 3 months following return from deployment (T2) plotted at different levels of pre-deployment neuroticism. The shaded area surrounding the line represents the 95% confidence interval. See Supplemental Figure 2a for an alternate visualization of the interaction.


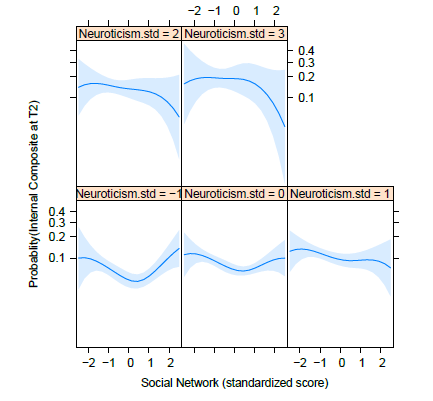


Supplemental Figure 3a

Association between pre-deployment low conscientiousness and probability of past 30-day distress disorders in the PPDS sample measured 3 months following return from deployment (T2) plotted at different levels of pre-deployment social network scores. The shaded area surrounding the line represents the 95% confidence interval. See Supplemental Figure 3b for an alternate visualization of the interaction.


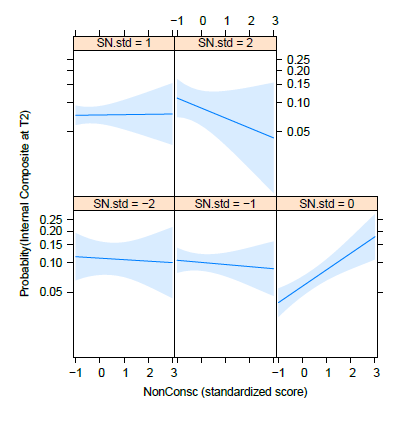


Supplemental Figure 3b

Non-linear association between perceived social networks measured pre-deployment and probability of past 30-day distress disorders in the PPDS sample measured 3 months following return from deployment (T2) plotted at different levels of pre-deployment low conscientiousness. The shaded area surrounding the line represents the 95% confidence interval. See Supplemental Figure 3a for an alternate visualization of the interaction.


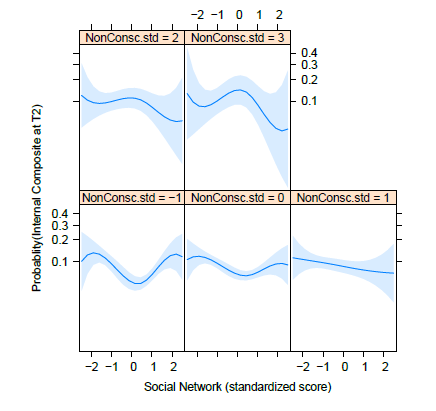


Supplemental Figure 4a

Association between pre-deployment neuroticism and probability of past 30-day suicidal behavior in the PPDS sample measured 9 months following return from deployment (T3) plotted at different levels of pre-deployment social network scores. The shaded area surrounding the line represents the 95% confidence interval. See Supplemental Figure 4b for an alternate visualization of the interaction.


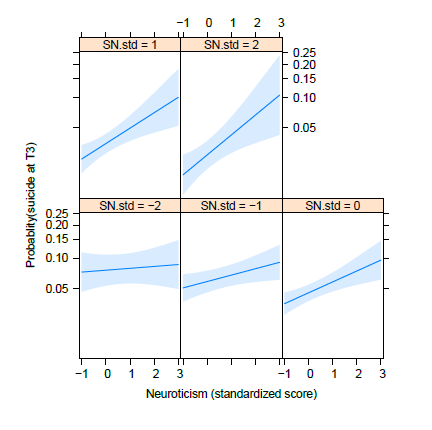


Supplemental Figure 4b

Association between pre-deployment social network size and probability of past 30-day suicidal behavior in the PPDS sample measured 9 months following return from deployment (T3) plotted at different levels of pre-deployment neuroticism scores. The shaded area surrounding the line represents the 95% confidence interval. See Supplemental Figure 4a for an alternate visualization of the interaction.


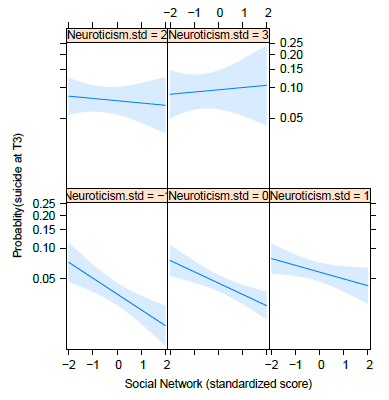

Supplement: Supplementary file 1 [file S0033291722002082sup001.docx]
